# A Sensitive, Specific and Simple Loop Mediated Isothermal Amplification Method for Rapid Detection of *Campylobacter* spp. in Broiler Production

**DOI:** 10.3389/fmicb.2019.02443

**Published:** 2019-10-24

**Authors:** Than Linh Quyen, Steen Nordentoft, Aaydha Chidambara Vinayaka, Tien Anh Ngo, Pia Engelsmenn, Yi Sun, Mogens Madsen, Dang Duong Bang, Anders Wolff

**Affiliations:** ^1^Department of Biotechnology and Biomedicine, Technical University of Denmark (DTU-Bioengineering), Lyngby, Denmark; ^2^National Food Institute, Technical University of Denmark (DTU-Food), Lyngby, Denmark; ^3^Department of Health Technology, Technical University of Denmark (DTU-Health Tech), Lyngby, Denmark

**Keywords:** campylobacteriosis, *Campylobacter* spp., loop mediated isothermal amplification, broiler fecal sample, broiler chicken production, rapid detection

## Abstract

Campylobacteriosis is one of the most common foodborne diseases worldwide. Two *Campylobacter* species – *C. jejuni* and *C. coli* in poultry and poultry products are considered to be the main source of human campylobacteriosis. Therefore, studying *Campylobacter* status in poultry flocks is needed to prevent transmission of disease and reduce human risk, health cost, and economic losses. In this study, we adapted and used a Loop-Mediated Isothermal Amplification (LAMP) assay for specific, sensitive, simple and cost-effective rapid detection of *C. jejuni* and *C. coli* in the poultry production chain. Amplified LAMP products were detected using a small, low-cost portable commercial blue LED transilluminator and a direct visual detection strategy was demonstrated. By using optimized conditions for amplification a limit of detection (LOD) of 50 CFU/ml was achieved for testing of *C. jejuni* and *C. coli* in spiked chicken feces without enrichment. The method took 60–70 min from receiving the samples to the final results (including 30 min for amplification). The optimized LAMP showed a relative accuracy of 98.4%, a specificity of 97.9%, and a sensitivity of 100% in comparison to real-time PCR method. Cohen’s kappa index also showed an excellent agreement (0.94) between the two methods. The results showed that the method is specific, sensitive and is suitable to develop for rapid detection of *Campylobacter* spp. at poultry production.

## Introduction

Campylobacteriosis is one of the leading causes of bacterial diarrhea worldwide (CDC, 2017; Helwigh et al., 2017). Two *Campylobacter* species, *C. jejuni* and *C. coli* account for the majority of human campylobacteriosis (Lawson et al., 1999; Tresse et al., 2017). Poultry and poultry products are considered to be the main sources for disease transmission (Wingstrand et al., 2006; Powell et al., 2012). The prevalence of *Campylobacter* in broiler flocks remains high (Sibanda et al., 2018). Data obtained from an electronically distributed survey in Denmark reported that 63% of the broiler farms tested positive for *Campylobacter* (Sandberg et al., 2015). Thus, there is an urgent need for a fast and simple method suitable for the detection of *Campylobacter* within poultry production chains.

Identification of *C. jejuni* and *C. coli* using conventional bacterial cultures in combination with biochemical-based assay are time-consuming (requiring more than 4 days) and laborious (Biesta-Peters et al., 2019). Therefore, several alternate methods have been developed and reported for the detection of *Campylobacter* spp. (Supplementary Table S1). Although ELISA (enzyme-linked immunosorbent assay) (RIDASCREEN^®^ Campylobacter, R-Biopharm AG, Darmstadt, Germany) and real-time polymerase chain reaction (real-time PCR) (Alves et al., 2016) could detect *Campylobacter* in much shorter time (within 2 h) from fecal materials, the limit of detection remains high (Supplementary Table S1). Moreover, real-time PCR require sophisticated and expensive equipment to amplify and detect the presence of *Campylobacter.* Therefore, both PCR and real-time PCR are not suitable for rapid detection of the pathogens in the production chains. LAMP has been used to overcome the drawbacks of the PCR. LAMP is faster than PCR (de Paz et al., 2014; Sabike and Yamazaki, 2019) and can be performed under constant temperature in a range of 60–65°C, thus eliminating the need for sophisticated thermal control as in PCR (Mori et al., 2013; Sun et al., 2015). LAMP has several advantages such as fast reaction, simple operation, low cost, high sensitivity and specificity (Njiru, 2012; Velders et al., 2018). Moreover, the LAMP reaction is more tolerant to inhibitors in comparison to PCR and real-time PCR assays (Stedtfeld et al., 2014; Kosti et al., 2015). The LAMP reaction produces large amounts of amplified products (dsDNA). It can therefore even be detected by naked eyes when using appropriate DNA staining techniques (Xie et al., 2014). With these advantages, the LAMP may be suitable for rapid detection of pathogens in the poultry production chains.

LAMP was developed for the detection of *Campylobacter* spp. in poultry samples such as meat, carcass swabs, and fecal samples (Yamazaki et al., 2008, 2009b; Sabike et al., 2016; Romero and Cook, 2018; Sabike and Yamazaki, 2019). To study the epidemiology of *Campylobacter* spp. and to prevent transmission in the production chain, the time taken for detection of *Campylobacter* is crucial. However, to detect the presence of the *Campylobacter* spp. in cloacal swabs, ceca, meat and environmental cleaning samples, an enrichment step of 22 to 24 h is needed (Yamazaki et al., 2009b; Sabike et al., 2016; Sabike and Yamazaki, 2019). Consequently, it takes a total of at least 24–26 h for sample enrichment, preparation, amplification, and detection. Moreover, *Campylobacter* grows slowly and requires specific microaerobic conditions to grow, which makes it difficult to apply this method for the detection of *Campylobacter* spp. in the production chains. In contrast, stool specimens may not require an enrichment step since *Campylobacter* infected chicken feces may contain up to 10^9^ *Campylobacter* per gram (Corry and Atabay, 2001; Hermans et al., 2010; Addis and Sisay, 2015). However, the content of inhibitors in feces is frequently high, which could inhibit the LAMP reaction (Schrader et al., 2012). In one study, to detect *Campylobacter* spp. directly from poultry feces with a limit of detection (LOD) of 1.2–1.4 CFU per test, the stool samples had to be diluted 1:4000 to reduce the inhibition effects (Yamazaki et al., 2008). Therefore, the method could not detect the fecal samples containing less than 1.2 × 10^6^ CFU of *C. coli* or 1.4 × 10^6^ CFU of *C. jejuni* per gram of stool specimen.

In this study, to provide a simple, rapid, cost-effective and sensitive method suitable for rapid detection of *Campylobacter* spp. within poultry production chains, we have developed an optimized LAMP assay with smaller reaction volume, a shorter reaction time, and higher sensitivity using a commercial LAMP kit. We also evaluated the use of a commercial mini UV transilluminator, which is small, simple, low cost and portable for LAMP detection. Moreover, for evaluation of the performance of the optimized LAMP method, a conventional real-time PCR was used in parallel to study the epidemiology of *Campylobacter* infection in a broiler farm.

## Materials and Methods

### DNA Preparation

Chromosomal DNA from all bacteria strains used in this study (listed in Table 1) was isolated using DNeasy Blood and Tissue kit (Qiagen, Germany). The DNA concentration was determined by NanoDrop (Thermo Scientific, United States).

**TABLE 1:** Bacterial strains used in this study.

**No.**	**Bacterial strains**	**Source**
1.	*C. jejuni*	NTCC 11284
2.	*C. coli*	CCUG 11283
3.	*C. lari*	CCUG 18267 and CUUG 860115
4.	*C. larilio 56*	CCUG 19512 and CUUG 920306
5.	*C. larilio 34*	CCUG 20575 and CUUG 870508
6.	*C. mucosalis*	CCUG 6822
7.	*C. sputorums* subsp. *spo*	CCUG 9728
8.	*C. upsaliensis*	CCUG 14913
9.	*C. upsaliensis*	CCUG 23626
10.	*C. fetus* subsq. *fetus*	CCUG 6823A and CCUG 940118
11.	*C. concisus*	CCUG 13144 and CUUG 950201
12.	*C. hyointestinalis*	CCUG 19512 and CUUG 920306
13.	*S.* Typhimurium	DVI Jeo 3979 Jgt.110
14.	*S.* Enteritidis	92243/nybol 3L
15.	*S.* Dublin	H64004
16.	*S. derby*	DVI SD1
17.	*E. faecalis*	ATCC 29212
18.	*E. faecium*	CCUG 47860
19.	*E. coli*	CCUG 17620
20.	*S. pneumoniae*	ATCC 49619
21.	*P. hauseri*	CCUG 36761
22.	*C. freundii*	CCUG 418
23.	*A. skirrowii*	CCUG 10374 and CCUG 910801
24.	*A. cryaerophilus*	CCUG 17801
25.	*A. butlezi*	CCUG30485
26.	*Y. ruckerii*	ATCC 29473

Preparation of DNA from fecal sock samples: Each pair of boot socks was placed in a stomacher bag containing 200 ml of saline (0.9% NaCl). Fecal materials were released by gentle manipulation of the socks for 2 min. 1 ml of the suspension was collected and centrifuged at 5000 × *g* for 5 min. After discarding the supernatant, the pellet was used for DNA extraction by an automate KingFisher^TM^ Purification system (Thermo Fisher, Copenhagen, Denmark) or stored at minus 20°C for later use. Further, the pellet was treated with 200 μl of lysis buffer consisting of 190 μl of magnetic lysis buffer and 10 μl of Proteinase K (20 mg/ml) followed by incubation for 10 min at room temperature. 100 μl of the treated sample was used to purify DNA by an automate KingFisher^TM^ Purification system using the Magnesil KF Genomic DNA kit (Promega, Denmark) described previously (Lund et al., 2003). Finally, the supernatant was used as the template for real-time PCR and the LAMP assay.

### Real-Time PCR

A real-time PCR targeting the 16S rRNA gene of thermophilic *Campylobacter* species (*C. jejuni*, *C. coli*) was used as a reference method to evaluate the performance of the LAMP (Josefsen et al., 2010). Primer sequences used for real-time PCR were listed in Supplementary Table S2. Each reaction contained 25 μl of master mixture consisting of 1X buffer, 2.5 mM of MgCl_2_, 2.5 U of Tth enzyme (Roche, Denmark), 6.96% Glycerol (Merck, Germany), 0.6 mM of dNTPs, 0.01 mg/ml of BSA, 0.5 μM of forward primer OT-1559, 0.5 μM of reverse primer 18-1, 0.076 μM of *Campylobacter* LNA probe, 0.06 μM of IAC probe, 0.24 × 10^–9^ μM of IAC (primers used for construction of the internal amplification control) (Supplementary Table S2) (Life Technology Europe, Roskilde, Denmark), 10 μl of DNA template and 2.22 μl of PCR grade water (Sigma-Aldrich, Denmark). The real-time PCR conditions were 95°C for 3 min, followed by 40 cycles of 95°C for 15 s, 60°C for 60 s and 72°C for 30 s. The real-time PCR was performed on a MxPro-Mx3005P (Agilent Technologies ApS Glostrup, Denmark).

### LAMP Assay

The LAMP assay was carried out using a Loopamp *Campylobacter* detection kit (Eiken Chemical Co., Ltd., Tokyo, Japan). The kit consists of a set of specific primers to recognize the oxidoreductase gene from *C. jejuni* and aspartate kinase gene from *C. coli*. The kit does not specifically identify *C. jejuni* or *C. coli* but can detect both species. LAMP reaction contained 12.5 μl of the master mixture consisting of 6.25 μl of the reaction mixture, 1.25 μl of primer mixture *Campylobacter*, 0.5 μl of *Bst* DNA polymerase, 2.5 μl of distilled water and 2 μl of the template. In the LAMP reactions, PCR grade water was used as negative control, and purified genomic DNA was used as positive control. The LAMP reaction was performed on a Dri-Block^®^ DB-2TC (TECHNE, Staffordshire, United Kingdom) at a constant temperature of 65°C for 30 or 60 min. The reactions were terminated by heating up to 80°C for 2 min.

### Preparation of *Campylobacter*-Spiked Fecal Samples for Testing the Sensitivity of LAMP

Initially, fecal sock samples were pre-confirmed for the absence of *C. jejuni* and *C. coli* by both conventional culture and real-time PCR. Different dilutions of *Campylobacter* cells for spiking in the fecal sock samples were prepared from serial 10-fold dilutions in saline water from stock cultures (OD_600_ = 0.3, spectrophotometer UV-1600PC) of a *C. jejuni* CCUG 11284 and a *C. coli* CCUG 11283 separately. 100 μl of each dilution was used to spike in 1 ml suspension of the *Campylobacter* negative fecal sock samples. The mixtures were centrifuged at 5000g for 5 min and the pellets were then used for DNA extraction as described above. The samples were analyzed by both LAMP and real-time PCR methods. Further, 100 μl of each dilution from 10^–1^ to 10^–8^ were spread on blood agar plates for determining colony forming unit (CFU) and the plates were incubated at 41.5°C in the microaerobic atmosphere. The CFU was determined by colonies counting after 48 h of incubation.

### Data Analysis

Evaluation of assays precision between real-time PCR and LAMP was calculated based on relative accuracy, relative specificity, relative sensitivity and Cohen’s kappa index as described previously (Chin et al., 2016; Vinayaka et al., 2019) using following formulas:

$$RelativeaccuracyAC\left(\%\right)=\frac{\left(\text{PA}+\text{NA}\right)}{\text{N}}\times 100$$

$$RelativespecificitySP\left(\%\right)=\frac{\text{NA}}{\text{N}-}\times 100$$

$$RelativesensitivitySE\left(\%\right)=\frac{\text{PA}}{\text{N}+}\times 100$$

$$C\,o\,h\,e\,n^{\prime }\,s\,K\,a\,p\,p\,a\,i\,n\,d\,e\,x=\,\frac{\text{P}\,\left(\text{o}\right)-\text{P}\,\left(\text{e}\right)}{1-\text{P}\,\left(\text{e}\right)}$$

Where:

PA: the positive agreement between the real-time PCR and LAMP methods;

NA: the negative agreement between the real-time PCR and LAMP methods;

N: total number of samples (NA + PA + PD + ND);

PD: false positives in the LAMP method;

ND: false negatives in the LAMP method;

N−: total number of negative results (NA + PD);

N+: total number of positive results (PA + PD);

P(o): (PA + NA)/N; and

P(e): {(positive recovery in real-time PCR/total number of tested samples (N)) × (negative recovery in real-time PCR/total number of tested samples (N))} + {(negative recovery in LAMP/total number of tested samples (N)) × (negative recovery in real-time PCR/total number of tested samples (N))}.

### Visual Detection of LAMP Products

LAMP products were visually detected by two different methods: direct visual detection under UV light by staining DNA using SYBR^®^ Safe DNA intercalating dye and agarose gel electrophoresis.

Direct visual detection using SYBR^®^ Safe staining DNA: The SYBR^®^ Safe (10 000X concentrate in DMSO, Life Technology, Denmark) was diluted 1:10, and 1 μl of diluted dye was added to each tube after LAMP reaction. The tubes were observed under UV light from a portable DR22 blue LED transilluminator (Supplementary Figure S1) (Clare Chemical Research, Inc., United States).

Gel electrophoresis detection: After LAMP reactions, 5 μl of each amplified LAMP product were loaded on 2% agarose gel containing 1X of SYBR^®^ Safe DNA Gel Stain (Invitrogen, Life Technologies, United States). Gel electrophoresis was carried out at 100 volts for 60 min and the gel electrophoresis patterns were observed under Bio-Rad Gel Doc 2000 UV transilluminator (Bio-Rad Life Science, Denmark).

### Study Design and Selection of Poultry Farm

**FIGURE 1:**
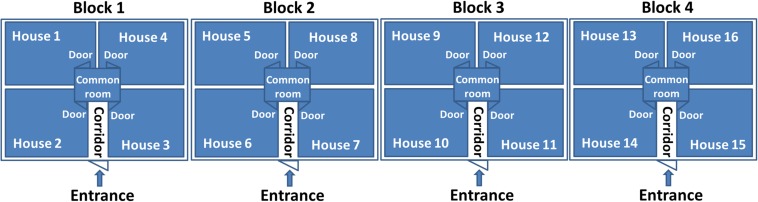
The design of the broiler houses.

To evaluate the ability of the method for rapid detection of *Campylobacter*, an evaluation trial was conducted in a large broiler farm located in Jutland, Denmark. The farm comprises 16 houses that are placed in blocks of four. All the four blocks have the same design with an entrance in the middle leading to a corridor from where there are doors to access to the 4 houses (Figure 1). During the poultry production period, fecal samples were collected from 16 houses, separately. Sampling was done on a weekly basis for four weeks by the boot sock sampling method as described previously (Food Standard Agency annual report, 2004/05). Briefly, elastic textile bands (Qualicum Scientific Ltd./Solar Biological Inc, United Kingdom) moistured with tryptone buffer (SteriSox, SODIBOX Nevez, France) were placed on clean boots. Fecal samples were collected from the floor of each poultry house by a farmer walking around the house. The socks were then placed in zipper bags and sent to the laboratory. The samples were processed in the lab as described above.

## Results and Discussion

### Optimization of LAMP Assay Conditions

**FIGURE 2:**
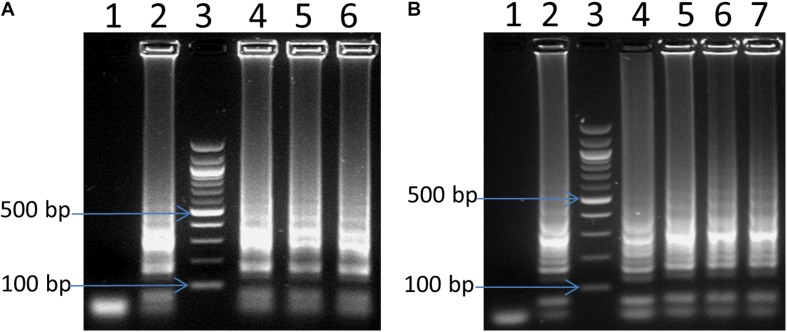
Optimization of LAMP for reaction volume **(A)** and amplification time **(B)**. In panel **(A)**, lane 4, 5 and 6: 6, 12.5, and 25 μL reaction volume, respectively. In panel **(B)**, lane 4, 5, 6 and 7: 15 min, 30 min, 45 and 60 min, respectively. In both panels **(A,B)**: lane 1: negative control, lane 2: positive control, and lane 3: 100 bp ladder.

Different volumes of the LAMP reaction ranging from 3 to 25 μl were selected and tested using 2 ng of *C. jejuni* DNA as a template. It is estimated that 2 ng DNA *C. jejuli* corresponds to 1.13 × 10^6^ genome equivalents (Chin et al., 2017). We observed LAMP products in all the reaction volumes tested such as 25, 12.5, and 6 μl (Figure 2A). Even small reaction volumes of 3 and 5 μL were sufficient enough to generate good LAMP signals (result not shown). The results indicated that the reaction volume had no influence on the LAMP efficiency and assay principle.

Different reaction times of 5, 10, 15, 30, 45, and 60 min using 2 ng of *Campylobacter* DNA as a template were also tested to define a suitable reaction time for the LAMP assay. There were no visible LAMP amplifications for 5 and 10 min reactions, but LAMP products were visible after 15 min (Figure 2B). More amplified LAMP products were obtained after 30 min, whereas the intensity remained the same as the reaction went on for 45 and 60 min (Figure 2B). Hence, 30 min of incubation has been selected for further experiments.

It is intended to adopt this technique into the poultry production chains wherein, the cost of each test is a primary concern. Many studies of LAMP assay were reported using different volumes (12.5 μl, 25 μl, even 50 μl) for LAMP reactions (Kato et al., 2005; Wang et al., 2008; Yamazaki et al., 2008, 2009b; Peng et al., 2011; Zhang et al., 2013). However, until now, no study has addressed appropriate reaction volume for LAMP assay. The cost of one *Campylobacter* Detection Kit with 48 reactions (25 μl per reaction as recommended by manufacture) from Eiken Ltd., Japan was 4456,50 DKK. As a consequence, each reaction (25 μl as recommended by the manufacturer) cost ∼93 DKK which was a rather high cost for screening pathogens at farms. Therefore, the result of this study showing reaction volume did not influence the efficiency of the assay will help reduction of the cost when using LAMP for the detection of pathogens at farms. Further, KingFisher^TM^ Purification system used in this study concentrates the samples and provides a good quality of DNA template. The cost of DNA extraction and purification is ∼23 DKK per sample and it is possible to process 24 samples at once in approximately 20 min.

Diagnosis time of foodborne disease screening is considered to be vital for preventing transmission and decreasing economic losses in broiler production. Rapid detection strategies with high specificity and sensitivity are of much relevance for broiler production. In this study, after 30 min of the LAMP reaction we were able to detect the presence of *C. coli*/*C. jejuni* in fecal samples as low as 50 CFU/mL without enrichment. The reaction time in this study was shorter in comparison to previous reports (35–60 min) (Iwamoto et al., 2003; Kato et al., 2005; Wang et al., 2008; Yamazaki et al., 2009a; Yang et al., 2010; Zhang et al., 2012, 2013). Zhuang et al. (2014) reported a LAMP reaction that can detect *Salmonella* in chicken feces within 25 min with a detection limit of 200 CFU per reaction that was 200 times higher than this study. Total time from sample preparation to the final result in this study was 60–70 min, and it is much shorter than previous reports (Kato et al., 2005; Okamura et al., 2008; Yamazaki et al., 2008, 2009b; Techathuvanan et al., 2010).

### Comparison of the Sensitivity of Optimized LAMP Assay at Different Reaction Times

**FIGURE 3:**
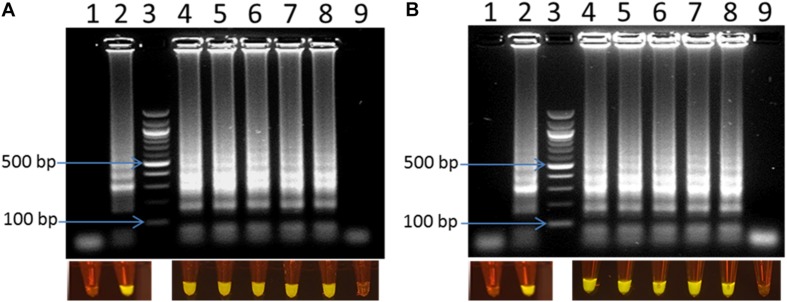
Visual detection of LAMP products on 2% agarose gel electrophoresis and using SYBR^®^ Safe staining DNA for sensitivity of the LAMP using pure DNA of *C. jejuni* in 30 min **(A)** and 60 min **(B)**. In both panels: lane 1: negative control, lane 2: positive control, lane 3: 100 bp ladder, and lane 4–9: 2 ng, 0.2 ng, 20 pg, 2 pg, 1 pg, 0.1 pg DNA of *C. jejuni*, respectively.

To compare the sensitivity of the optimized LAMP assay, reaction time of 30 min was compared with 60 min of amplification. A serial dilution of *C. jejuni* DNA ranging from 0.1 pg to 2 ng was prepared and used as a template for the LAMP reaction. Figure 3 shows that there were no LAMP amplified products when 0.1 pg of DNA template was used in both reaction times. In contrast, LAMP amplified products were observed with all other template concentrations tested. This result confirmed that, the optimized LAMP has similar sensitivity at 30 and 60 min of amplification.

### Sensitivity of Optimized LAMP Assay in *C. jejuni/C. coli* Cells Spiked Fecal Samples

**FIGURE 4:**
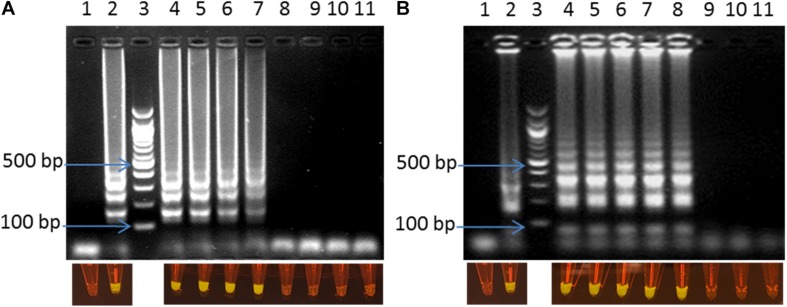
Visual detection of LAMP products on 2% agarose gel electrophoresis and using SYBR^®^ Safe staining DNA for the sensitivity of the fecal spiked sample with *C. jejuni* **(A)** and *C. coli* **(B)**. In both panels: lane 1: negative control, lane 2: positive control, lane 3: 100bp ladder, and lane 4–11: 10^–1^–10^–8^ dilution.

The optimized LAMP assay was also tested with the whole cell of *C. jejuni* and *C. coli* spiked into chicken fecal samples as described above. LODs of 1 CFU/reaction (corresponding to 50 CFU/ml) were observed for both *C. jejuni* and *C. coli* within 30 min of amplification (Figure 4). The optimized LAMP assay, in this study, showed better performance with lower LOD and shorter reaction time than previous reports (1.2–1.4 CFU and 5 CFU per reaction within 60 min) (Yamazaki et al., 2008; Romero and Cook, 2018).

### Application of the Optimized LAMP Assay to Detect *Campylobacter* in Poultry Farm

**FIGURE 5:**
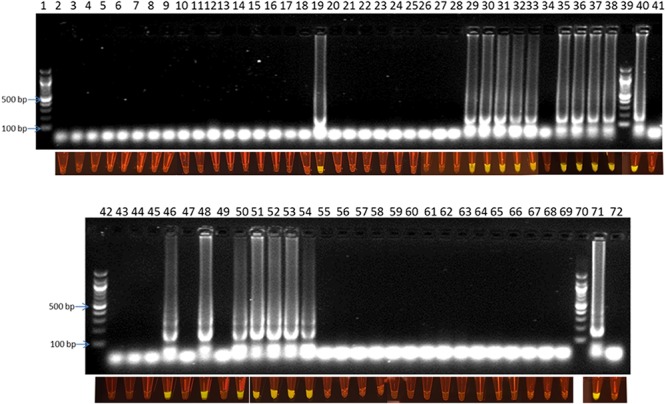
Visual detection of LAMP products on 2% agarose gel electrophoresis and using SYBR^®^ Safe staining DNA for chicken fecal samples: lane 1, 39, 42, and 70: 100 bp ladder, lane 40 and 71: positive control, lane 41 and 72: negative control, and lane 2–38 and 43–69: samples from rotation 1, 2 and 3.

Efficiency of the optimized LAMP assay was also tested with real poultry fecal samples. A total of 64 boot-sock samples were collected and tested for the presence of C. *jejuni* and *C. coli*. Results showed that out of 64 samples tested, 17 samples were positive and 47 samples were negative for *C. jejuni* and *C. coli* (Table 2). There was 100% coincidence between the gel electrophoresis-based detection method and the visual observation using blue LED transilluminator (Figure 5). On the other hand, in the real-time PCR method, 18 samples were found positive and 46 samples were found negative with *C. jejuni*/*C. coli*. Comparing the results of the two methods showed a relative accuracy, specificity, and sensitivity of 98.43, 97.87, and 100%, respectively for the LAMP method. Cohen’s kappa index showed an excellent agreement between the real-time PCR and the LAMP (Cohen’s kappa = 0.94) (Table 2). One sample tested positive with the real-time PCR but negative with LAMP assay was further confirmed by conventional PCR using a different primer set developed in our lab. Out of four tests performed with this sample, the results were negative for *C. jejuni*/*C. coli* in 2 attempts and positive only for *C. jejuni* in another 2 attempts (data not shown). This difference in the results between the two methods may be attributed to a low number of target concentration in the tested sample.

**TABLE 2:** Comparison of real-time PCR and LAMP assay for detection of chicken feces from sock samples.

**Samples**	**Real-time PCR**	**LAMP**
Positive	18	17
Negative	46	47
Total	64	64
**Comparison of real-time and LAMP**		
Relative accuracy (AC%)	98.43	
Relative specificity (SP%)	97.87	
Relative sensitivity (SE%)	100	
Cohen’s kappa index	0.94	

### Visual Detection of LAMP Products Using Commercial Portable DR22 Blue LED Transilluminator

LAMP products could be detected by gel electrophoresis after amplification. Figure 3 showed that positive amplification was observed in positive control and with DNA target from 1 pg to 2 ng. On the other hand, the products could also be observed directly by adding 1 μl of 1:10 SYBR^®^ Safe. Under a commercial portable DR22 blue LED transilluminator, both positive control and the reactions with DNA target concentrations ranging from 1 pg to 2 ng of DNA changed from transparent to yellow, while no color change was observed in the negative control and with 0.1 pg of DNA template (Figure 3). This result showed that there was no difference in the detection limit between the gel electrophoresis and color-change observation by the low cost commercial DR22 blue LED transilluminator. The coincidence of results of gel electrophoresis and direct visual detection were also observed in Figures 4, 5.

### Determination of Specificity of the LAMP Assay

Twenty six bacterial strains as listed in Table 1 were tested for the specificity of the assay. As one can see in Supplementary Figure S2, LAMP positive reactions were observed only with *C. jejuni* and *C. coli* DNA templates, while no LAMP amplification was detected from the reactions using DNA from the other 24 bacterial reference strains that included 10 other *Campylobacter* species. The results showed that the LAMP assay was highly specific for *C. jejuni* and *C. coli*.

### Epidemiology of *Campylobacter* in Broilers Farm

For this study, we have collected fecal samples weekly from sixteen broiler houses for testing *Campylobacter* through three rotations of poultry production (Table 3). After each rotation, the houses were cleaned and disinfected before starting a new rotation. The first rotation lasted 4 weeks. In the first and second week, *Campylobacter* was not detected in any of the sixteen houses. In the third week, *Campylobacter* was found in house number 2 in block 1 and house 16 in block 4. Only a week later, *Campylobacter* was detected in all the houses of block 1 and block 4. The second rotation lasted only 2 weeks. In the first week, *Campylobacter* was detected in house number 14 and 16 of block 4. One week later, *Campylobacter* was found in all the houses in block 4 and also house number 12 in block 3. The third rotation in this study lasted four weeks and no *Campylobacter* was found in the first and second week. In the third week, *Campylobacter* was found in house number 12 in block 3. In the fourth week, all houses in block 3 were positive for *Campylobacter*. The results showed clearly that, once *Campylobacter* was introduced into the broiler houses, it was transmitted easily and quickly to other broilers houses and other broilers blocks.

**TABLE 3:** Screening of *Campylobacter* from rotations.

	**House**	**Rotation 1**				**Rotation 2^∗^**		**Rotation 3**			
		**Week 1**	**Week 2**	**Week 3**	**Week 4**	**Week 1**	**Week 2**	**Week 1**	**Week 2**	**Week 3**	**Week 4**
Block 1	1	−	−	−	+	−	−	−	−	−	−
	2	−	−	+	+	−	−	−	−	−	−
	3	−	−	−	+	−	−	−	−	−	−
	4	−	−	−	+	−	−	−	−	−	−
Block 2	5	−	−	−	−	−	−	−	−	−	−
	6	−	−	−	−	−	−	−	−	−	−
	7	−	−	−	−	−	−	−	−	−	−
	8	−	−	−	−	−	−	−	−	−	−
Block 3	9	−	−	−	−	−	−	−	−	−	+
	10	−	−	−	−	−	−	−	−	−	+
	11	−	−	−	−	−	−	−	−	−	+
	12	−	−	−	−	−	+	−	−	+	+
Block 4	13	−	−	−	+	−	+	−	−	−	−
	14	−	−	−	+	+	+	−	−	−	−
	15	−	−	−	+	−	+	−	−	−	−
	16	−	−	+	+	+	+	−	−	−	−

*−: negative; +: positive. ^∗^Rotation 2 last only 2 weeks since once *Campylobacter* was introduced to the broiler houses; it is transmitted easily and quickly to other broiler houses. So producer decided to stop the production in order to reduce economic loss.*

*Campylobacter* is abundant in cloaca, cecum and large intestine of poultry, up to 10^9^ CFU/g of feces (Corry and Atabay, 2001; Hermans et al., 2010). After excretion, *Campylobacter* survives from a minimum of 2 days up to 14 days in the feces (Smith et al., 2016). Therefore, the feces may play a key role in the transmission of *Campylobacter*. Wild animals such as crawling insects, arthropods and flies have been shown to be vectors that can transfer *Campylobacter* from outside into broiler houses as well as from fecal materials inside broiler houses to other broiler houses (Sheppard, 2014). A study carried out in Denmark showed that there were 8.2 and 70.2% of flies caught outside a broiler house positive with *Campylobacter* as confirmed with conventional culture and PCR method, respectively (Hald et al., 2004). This study suggests that flies may play an important role in *Campylobacter* infection of broiler flocks during summer. Besides, air and dust are also considered as a source of the transmission (Berrang et al., 2004; Wilson, 2004). Bull et al. (2006) reported that approximately 6% of air samples were positive for *Campylobacter* once the broiler flocks were positive with *Campylobacter* (Bull et al., 2006). Furthermore, in the early stage of the colonization, *Campylobacter* can spread into the air of the broiler house (Olsen et al., 2009).

In addition, water may also contribute as a source of *Campylobacter* transmission in broilers houses. Bull et al. (2006) reported that approximately 31% of water samples from drinkers were positive with *Campylobacter* once flocks were positive with *Campylobacter* (Bull et al., 2006). Moreover, *Campylobacter* can persist for long periods in well-water and can also survive under various conditions for days (Buswell et al., 1998), weeks (Korhonen and Martikalnon, 1991), and even months (Rollins and Colwell, 1986; Thomas et al., 2002) in different aqueous environment (Schallenberg et al., 2005).

Although humidity in the broiler house is not a direct source of the transmission, it can influence the transmission of *Campylobacter*. Line (2006) showed that, at low relative humidity (30% ± 10%), the colonization of *Campylobacter* was delayed compared with high relative humidity (80% ± 10%) since it has been shown that water can enhance the survival of *Campylobacter* in broiler flocks (Trigui et al., 2015).

## Conclusion

In summary, the present study describes a simple and rapid LAMP assay for the detection of *C. jejuni* and *C. coli* in chicken feces. The assay conditions were optimized for low reaction volume and shorter time of reaction. With the optimized conditions, it was possible to detect *C. jejuni* and *C. coli* in spiked chicken feces as low as 50 CFU/ml within 60–70 min in total. The LAMP assay was compared with an in house real-time PCR. Cohen’s kappa index showed excellent agreement between the two methods. The optimized LAMP method was used to study the transmission of *Campylobacter* at a Danish poultry farm. The results confirmed the capability of the LAMP technique as a rapid screening method for the detection of *Campylobacter* spp. at poultry production.

## Data Availability Statement

All datasets generated for this study are included in the manuscript/Supplementary Files.

## Author Contributions

DB, AW, AV, SN, MM, and YS designed the work. TQ and PE performed the experiments. DB, TQ, AW, AV, SN, TN, and YS wrote the manuscript.

## Conflict of Interest

SN is currently employed by the company Novo Nordisk A/S, Bagsværd, Denmark. TN is currently employed by Vinmec HealthCare System, Hanoi, Vietnam. The remaining authors declare that the research was conducted in the absence of any commercial or financial relationships that could be construed as a potential conflict of interest.
