# Supplementary material for: A Sensitive, Specific and Simple Loop Mediated Isothermal Amplification Method for Rapid Detection of Campylobacter spp. in Broiler Production
Source: Front Microbiol. 2019 Oct 24;10:2443. doi: 10.3389/fmicb.2019.02443 (PMC6821646; doi:10.3389/fmicb.2019.02443)

Supplementary Material

**Table S1.** Detection methods of *Campylobacter* spp.

| **Method** | **Matrix** | **Enrichment time (hours)** | **Total analysis time (min)** | **LOD** | **Specificity (%)** | **Reference** |
| --- | --- | --- | --- | --- | --- | --- |
| **ELISA** | Human feces | No | 50 | 10^4^-10^5^ CFU/ml | 100 | Food Campylobacter Antigen ELISA Test Kit, Diagnostic automation INC, USA |
|  | Human feces | No | 60-105 | 1.9x10^4^ - 1.1x10^6^ CFU/ml | 99.2 | ELISA RIDASCREEN® Campylobacter (R-Biopharm AG, Darmstadt, Germany) |
| **Lateral flow^1^** | Food products | 24 |  | 50 CFU/ml (detected by SERS) | 100 | Duyn He et al. 2019 |
|  | Milk sample | No |  | 75 CFU/ml (detected by color mode) | 100 | Duyn He et al. 2019 |
| **qPCR** | Chicken meat | No | >115 | 10^3^ CFU/ml | 100 | Juliane Alves et al. 2016 |
|  |  | 24 | >115 | 1 CFU/ml | 100 |  |
|  | Feces | No | >100 | 2.5x10^2^ CFU/g | 96.2 | Leblanc-Maridor et al., 2011 |
|  | Feed | No | >100 | 1.3x10^2^ CFU/g | 96.2 |  |
|  | Environmental sample | No | >100 | 1.0×10^3^ CFU/m^2^ | 96.2 |  |
| **LAMP** | Poultry sample | No | 65 | 10^4^ CFU/swab | - | Romero and Cook, 2018 |
|  | Chicken cloacal swab | No | 90 | 3.89-3.60 log CFU/g | 100 | Sabike et al., 2016 |
|  | Chicken feces | No | 60 | 50 CFU/ml | 100 | This study |

^1^When using color mode, the intensity of the color in positive reaction was very poor. Therefore, it is difficult to differentiate at LOD lower than 10^5^CFU/ml.

**Bacterial strains and culture condition**

26 Campylobacteriaceae and related organisms used in this study (listed in Table 1) were obtained from a culture collection of National Food Institute, Technical University of Denmark (DTU-Food). The NCTC strains originated from the National Collection of Type Cultures, and the CCUG strains originated from the Culture Collection at the University of Gothenburg. All the strains were stored at -80 ^o^C in microorganism preservation system (Technical Service Consultants Ltd, Microbiology House, Fir Street, Heywood, Lancashire OL10 1NW, UK). The *Campylobacter* strains were resuscitated and selected on Charcoal Cefoperazone Deoxycholate Agar (CCDA (Thermo Fisher Scientific, Denmark) and grown on blood agar (BA) (Statens Serum Institute, Copenhagen, Denmark) at 41.5 °C for 48 hours under microaerobic conditions (5% O2, 10% CO2, in 85% N2). *Salmonella* strains, *Enterococcus faecalis (E. faecalis),* and *Enterococcus faecium (E. faecium)*, *Escherichia coli (E. coli)*, *Streptococcus pneumoniae (S. pneumoniae)*, *Proteus hauseri (P. hauseri),* and *Citrobacter freundii (C. freundii)* were grown on BA at 37 °C overnight in aerobic conditions. *Arcobacter* strains were grown on BA in aerobic conditions at 15 °C or room temperature.

**Table S2.** Primer sequences for real-time PCR used in this study.

| **Name** | **Primer sequences (5’-3’)** |
| --- | --- |
| OT-1559 (forward primer) | CTGCTTAACACAAGTTGAGTAGG |
| 18-1 (reverse primer) | TTCCTTAGGTACCGTCAGAA |
| LNA probe | (6FAM)CA(+T)CC(+T)CCACGCGGCG(+T)TGC(BHQ1) |
| IAC probe | Joe-TTCATGAGGACACCTGAGTTGA-Tamra |
| IAC | CTGCTTAACACAAGTTGAGTAGGCAACTCAGGTGTCCTCATGAATTTGAAGACATAAACAAGGGACTGGTCTCCGTCCCAACCAAGATCATCCATCTCCCGCTATTCTGACGGTACCTAAGGAA |

**Figure S1**. A hand held DR22 blue LED transilluminator. The transilluminator has dimensions of 16x4.5x7 cm and weight approximately 0.45kg.


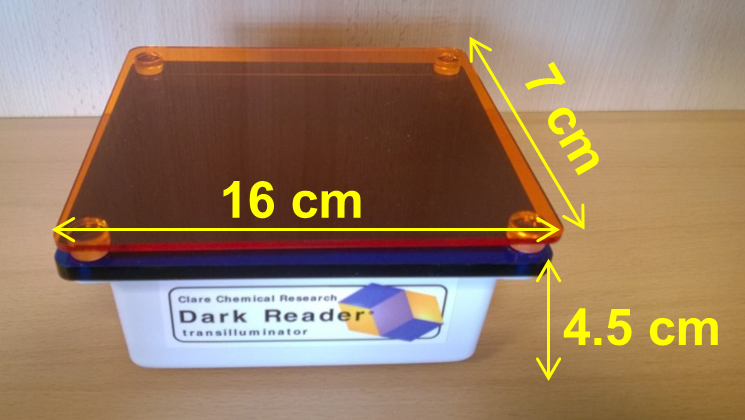


**Figure S2.** Testing the specificity of the primers by LAMP. Lane 1: negative control; lane 2: positive control; lane 3: 100bp ladder; lane 4 - 29: *C. jejuni*, *C. coli*, *C. lari*, *C. lari lio* 56, *C. lari lio* 34, *C. mucosilis*, *C. sputorum ss.spo*, *C. upsaliensis*, *C. upsaliensis*, *C. fetus* subsq. *Fetus*, *C. concisus*, *C. hyointestina*, *S. typhimurium*, *S. enteritis*, *S. dublin*, *S. derby*, *E. faecalis*, *E. faecium*, *E. coli*, *S. pneunomiae*, *P. hauseri*, *C. freundii*, *A. skirrowii*, *A. cryaophilus*, *A. butlezi*, *Y. ruckerii*, respectively.
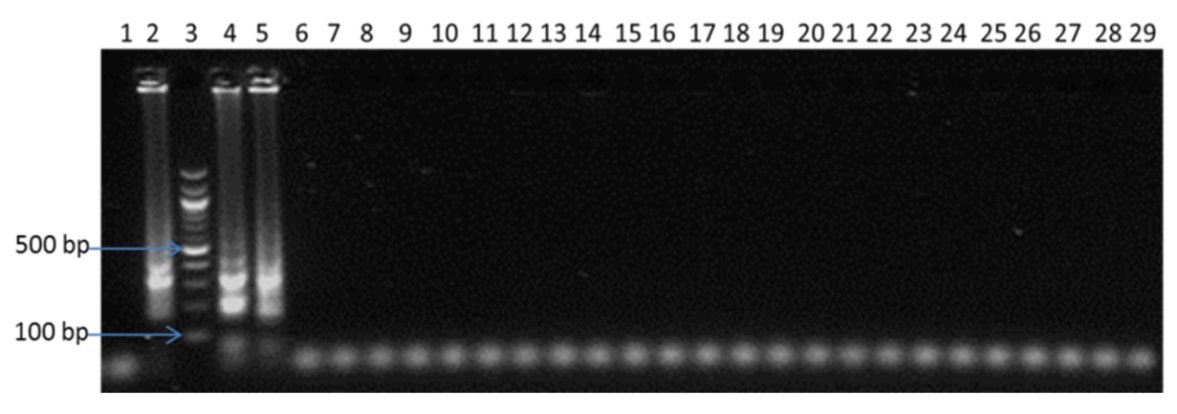

Supplement: Supplementary file 1 [file Data_Sheet_1.docx]
